# An Online Evidence-Based Education Resource Is Useful and Can Change People’s Perceptions About Running and Knee Health

**DOI:** 10.2519/josptopen.2024.0149

**Published:** 2024-06-21

**Authors:** Manuela Besomi, Michael A. Hunt, Danilo de Oliveira Silva, Samuele Passigli, Michael Skovdal Rathleff, Marienke van Middelkoop, Christian Barton, Michael J. Callaghan, Matthew S. Harkey, Alison M. Hoens, Natasha M. Krowchuk, Anthony Teoli, Bill Vicenzino, Richard W. Willy, Jean-Francois Esculier

**Affiliations:** 1 School of Health and Rehabilitation Sciences, The University of Queensland, Brisbane, Australia.; 2 The Running Clinic, Lac-Beauport, Québec, Canada.; 3 School of Physical Therapy, Universidad del Desarrollo, Santiago, Chile.; 4 Department of Physical Therapy, University of British Columbia, Vancouver, British Columbia, Canada.; 5 Motion Analysis and Biofeedback Laboratory, University of British Columbia, Vancouver, British Columbia, Canada.; 6 La Trobe Sport and Exercise Medicine Research Centre (LASEM), School of Allied Health, La Trobe University, Melbourne, Australia.; 7 University of Rome Tor Vergata, Rome, Italy.; 8 Center for General Practice at Aalborg University, Aalborg University, Aalborg, Denmark.; 9 Department of Occupational Therapy and Physiotherapy, Aalborg University Hospital, Aalborg, Denmark.; 10 Department of Health Science and Technology, Faculty of Medicine, Aalborg University, Aalborg, Denmark.; 11 Department of General Practice, Erasmus MC Medical University, Rotterdam, The Netherlands.; 12 Department of Physiotherapy Podiatry and Prosthetics and Orthotics, School of Allied Health, Human Services and Sport, La Trobe University, Bundoora, Australia.; 13 Department of Health Professions, Manchester Metropolitan University, Manchester, UK.; 14 Manchester University Foundation NHS Trust, Manchester, UK.; 15 Department of Kinesiology, Michigan State University, East Lansing, MI.; 16 Arthritis Research Canada, Vancouver, British Columbia, Canada.; 17 Centre for Health Evaluation and Outcome Sciences, University of British Columbia, Vancouver, British Columbia, Canada.; 18 School of Physical & Occupational Therapy, McGill University, Montreal, Québec, Canada.; 19 Lethbridge Layton Mackay Rehabilitation Center, Montreal, Québec, Canada.; 20 InfoPhysiotherapy, Montreal, Québec, Canada.; 21 School of Physical Therapy, University of Montana, Missoula, MT.; 22 MoveMed Physiotherapy, Kelowna, British Columbia, Canada.

**Keywords:** education, joint health, knee osteoarthritis, running, survey

## Abstract

**OBJECTIVES:**

To (1) create and evaluate the usefulness of an online evidence-based education resource about running and knee health (eg, knee osteoarthritis) for the public and health care professionals, and (2) assess the online resource’s effects on perceptions about running and knee health.

**DESIGN:**

Cross-sectional survey.

**METHODS:**

We created an online education resource (series of infographics) in 7 languages with community input. Then, we conducted a single-round online survey in which participants rated its usefulness and answered questions on perceptions about running and knee health before and after reading the infographics.

**RESULTS:**

Two thousand six hundred ninety-four participants (1291 members of the general public and 1403 health care professionals; 45.7% with knee osteoarthritis and 67.6% runners) from 60 countries viewed the infographics and responded to the postinfographics questions. The online resource was considered very useful, with a median rating of 9 out of 10. 23.2% of participants reported no change in their perceptions about running and knee health, 46.2% changed a little bit, 19.3% changed a moderate amount, and 11.3% changed a lot. Perceptions of running were more favorable after reading the infographics, especially about the effects of regular and frequent running on knee health, and running in individuals with knee osteoarthritis. Perceptions about running long distances were less favorable after the infographics.

**CONCLUSION:**

Our free online education resource was considered useful by both the public and health care professionals. Overall, the online resource led to more positive perceptions about recreational running and knee health. However, its effects on behavior change and running participation remain unknown.

Running is one of the most accessible and popular physical activities globally, and throughout the lifespan. It has many known benefits on physical^28,37^ and psychological^43^ health, but many people are unaware of the effects with regards to joint health.^17,19^ In a survey conducted with the general public and health care professionals in Canada, 1 in 3 respondents were uncertain if running frequently was detrimental for the knee joint.^19^ Close to half were uncertain when asked about long-distance running and knee joint health.^19^ In a follow-up international multilingual survey, half of the 4521 respondents perceived that running long distances increased the risk of developing knee osteoarthritis (OA).^17^ Given that running is a popular form of exercise and that exercise is universally recommended for managing knee OA,^3,6,33,43,46^ uncertainty may preclude some individuals from continuing to run as they age or after a diagnosis of knee OA.

Research does not definitively support the association between recreational running and knee OA.^2^ Sedentary individuals (nonrunners) have 3 times more knee and hip OA than recreational runners.^1^ A history of running could even be protective, and has been associated with 54% lower odds of requiring surgery due to knee OA.^45^ Competitive running, however, has been linked with a higher prevalence of knee OA,^1^ similar to other competitive sports—even weight-lifting.^11^ Runners and clinicians may feel uncertain about whether running is good for knee health, yet current evidence suggests it is safe when practiced recreationally. Even though recommendations may become more specific when more research is published, negative perceptions are likely not due to an evidence gap, but rather a knowledge translation gap.

Education resources are needed to provide current and accurate information about running and knee health.^14^ Educating the public, especially about self-management strategies to promote self-efficacy,^36^ can positively impact physical and psychological outcomes in individuals affected by chronic health conditions.^34,40^ In people with knee OA, evidence-based education combined with exercise therapy effectively improves pain and function.^25^ Disseminating the latest evidence can lead to changes in perceptions, hopefully leading to behavior change.^34^

It is important to recognize that behavior change is complex and influenced by a wide variety of factors beyond knowledge alone. While knowledge is a necessary component, it is only 1 piece of a larger puzzle in promoting lasting behavior change. Individuals who fear damaging their joints may resume recreational running after reading about its potential benefits on joint health. Similarly, health care professionals may shift to more positive clinical recommendations toward running if current myths are dispelled. The objectives of this study were to (1) create and evaluate the usefulness of an online evidence-based educational resource about running and knee health for the public and health care professionals, and (2) assess the effects of the online resource on perceptions about running and knee health.

## METHODS

This investigation is the second part of a previously published online survey.^17^ The first part was a cross-sectional survey that compared the perceptions of the general public and health care professionals about running and knee health and explored recommendations provided by health care professionals. The present investigation was a single-round open survey focusing on the usefulness of an online educational resource and changes in perceptions about running and knee health. It is reported according to the Checklist for Reporting Results of Internet E-Surveys.^20^ Ethics approval was obtained from the Behavioural Research Ethics Board at the University of British Columbia (H19–03859).

### Participants

Anonymous responses (convenience sample) were collected between June 18 and October 1, 2020. To be included, participants had to be aged 18 years and older; have Internet access; and understand English, French, Spanish, Portuguese, Dutch, Danish, or Italian. Participants were recruited through social media (paid advertisements; channels of the coauthors and their collaborators), sports, and arthritis interest groups and email lists.

Potential participants selected their preferred language, read the study objectives, and expected survey time, and provided informed consent electronically. Thereafter, they self-identified as members of the general public or health care professionals and were directed to the corresponding questions on demographics and perceptions about running and knee health. No incentives were provided.

### Survey and Online Educational Resource

Designing the survey questions consisted of 4 steps, described in more detail elsewhere^17^: (1) designing the first English version, (2) iteratively refining the survey questions with members of the general public to ensure that the survey was measuring what we intended to measure, (3) translating the survey and getting feedback on each translated version from members of the public and health care professionals, and (4) administering the survey online (Qualtrics XM, SAP America).

The same 4 steps were used to develop an online educational resource about knee OA and running, based on the latest evidence available at the time, including (1) description and epidemiology of knee OA,^48^ (2) association between imaging and symptoms,^10,22^ (3) risk factors for knee OA,^11,39^ (4) benefits of running on overall health,^13,28,31,37,38,43^ (5) association between running and knee OA prevalence,^1,45^ (6) benefits of running on knee health in healthy individuals,^29,30,47^ (7) running in people with knee OA and suggested training modifications,^5,18,35^ and (8) a summary infographic. A title page and references were also provided, for a total of 10 infographics. Members of the public, including runners and nonrunners with and without knee OA, as well as health care professionals provided feedback on the questions and online resource. Based on feedback, changes were made to the vocabulary, explanations, and visual appeal to reach the final version. The online educational resource is freely available online, in all 7 languages (http://hdl.handle.net/2429/82767).^16^

### Data Collection

After completing the initial survey questions,^17^ participants were offered the opportunity to view an educational resource on running and knee health. Participants who did were then asked to answer more questions (TABLE 1), including their perception of the online resource’s usefulness (Q1; 0 to 10, sliding scale with 1 decimal, where 0 means “not useful at all” and 10 means “very useful”), to what degree (if any) the online resource changed their perceptions about running and knee health (Q2), and if so, 7 questions (Q3-Q9) about their perceptions. These 7 questions were the same as before viewing the online resource, and identified if participants’ perceptions were positive, neutral, or negative about certain aspects of running and knee health. Participants also identified what they believed should be priorities for future research (Q10). Those who did not view or chose not to answer questions after the online resource were brought to the end of the survey, and their data were not considered in these analyses. The order of questions or items was not randomized. Adaptive questioning (branching) was used when required. All questions were mandatory and checked by the online system. Participants were allowed to go back to review and change their responses; however, they did not have an opportunity to go back and change their preinfographics responses after viewing the infographics.

The survey used a skip logic function; respondents who declared that their perceptions changed after the infographics were directed to Q3-Q9, whereas those who indicated no change in perceptions had their preinfographics responses duplicated for the purpose of analysis. All participants who viewed the online resource and rated its usefulness (Q1) were included in this study and considered for Objective 1. Those who answered about changes in perceptions after viewing the online resource (Q2) were considered for Objective 2.

### Data Analysis

Both the preinfographics and postinfographics data were exported to a Microsoft Excel spreadsheet. As multiple health care professionals from the same workplace, or members of the same family could have participated, we did not exclude multiple responses from the same IP address. Descriptive data were computed for all questions.

Responses to questions about perceptions were grouped to provide a general estimate of change in perception (eg, “inappropriate” included both “somewhat inappropriate” and “very inappropriate”) resulting in 4 main categories of perceptions (positive, neutral, negative, and “I don’t know”). Preinfographics and postinfographics proportions were compared using chi-square tests (2 × 4 contingency tables). Effect sizes (Cramer V, 3 degrees of freedom) were categorized as negligible (<0.06), small (≥0.06), moderate (≥0.17), or large (≥0.29).^8,32^ An alpha level of 0.05 was chosen, and Bonferroni adjusted (*P*<.0125) based on 4 response categories. We also conducted an exploratory subgroup analysis, comparing the change in perceptions between runners and nonrunners, as well as between participants with and without knee OA. All statistical analyses were conducted in Stata 17.^42^

### Patient and Public Involvement

Members of the general public, including individuals with knee OA, provided feedback when designing survey questions and the educational resource in each of the 7 languages. Participants also provided input on research priorities, to help ensure future studies address knowledge users’ needs.^15^

### Equity, Diversity, and Inclusion

Our research team included more men than women, from different stages of career ( junior to senior researchers). The 7 languages used all originated from Europe but are also spoken in various parts of the world. They were chosen based on the primary languages spoken by our team members. Running is a highly accessible and cost-effective physical activity, regardless of cultural background or socioeconomic status, and more efforts could have been made to stimulate participation from marginalized groups, including individuals from low-income countries and people with lower levels of education. The project leaders reached out to running initiatives for Indigenous communities in British Columbia to promote the study. Demographic questions were gender inclusive but did not ask about ethnicity.

## RESULTS

Out of the 4521 participants who answered the preinfographics questions,^17^ 2694 (59.6%) participants from 60 countries agreed to view the online resource (1120 in English, 398 in French, 385 in Spanish, 303 in Portuguese, 275 in Italian, 159 in Danish, and 54 in Dutch) (SUPPLEMENTAL FIGURE S1). Our sample included 1291 members of the general public and 1403 health care professionals (TABLE 2). After reading the online resource, 206 responded to the usefulness question only—the remaining 2488 continued to complete the survey.

### Objective 1: Usefulness of the Online Educational Resource

The online resource was considered useful to learn about running and knee health, with a median score of 9.0 (interquartile range [IQR], 8.0–10.0). Both the general public (median, 9.1; IQR, 8.0–10.0) and health care professionals (median, 9.0; IQR, 7.6–10.0) considered it useful to educate their peers. Responses to preinfographics questions did not discriminate which participants found the online resource more useful. Ratings of usefulness were similar (*P*>.05) between those who only responded to the usefulness question (n = 206; median, 9.1; IQR, 8.0–10.0) and those who answered all the postinfographics questions (n = 2488; median, 9.0; IQR, 8.0–10.0).

### Objective 2: Changes in Perceptions

After reading the online resource, 578 (23.2%) participants said that their perceptions about running and knee health did not change at all, while 1150 (46.2%) changed a little bit, 479 (19.3%) changed a moderate amount, and 281 (11.3%) changed a lot. Change in perceptions represents all changes regardless of the direction (positive/negative).

**FIGURE 1:**
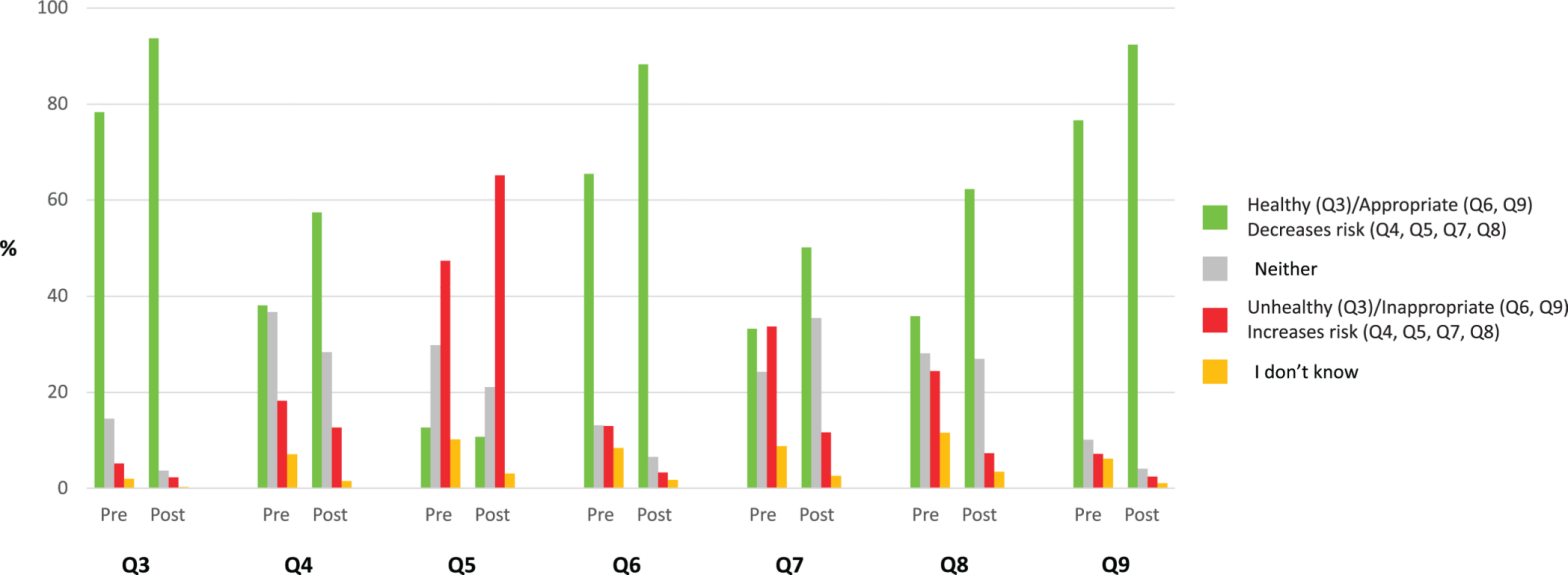
Proportions of pre-educational and posteducational resource responses in the overall population (general public and health care professionals).

Proportions of preinfographics and postinfographics responses to Q3 to Q9 are presented in FIGURE 1.

Perceptions about the effects of regular running (at least once per week), frequent running (at least 3 times per week), and running long distances (such as marathons and ultramarathons) on knee health, and about running in individuals with knee OA were different between the preinfographics and postinfographics questions (*P*<.001), with moderate to large effect sizes (Cramer V = 0.21–0.32) (SUPPLEMENTAL TABLE S2).

After reading the infographics, a greater proportion of participants perceived regular running as healthy for knees compared with preinfographics responses (93.7% vs 78.1%; χ^2^ [df = 3; n = 2488] = 258.32; *P*<.001; Cramer V = 0.23). More participants perceived that running frequently decreased the risk of developing knee OA (57.4% vs 38.6%; χ^2^ [df = 3; n = 2488] = 227.91; *P*<.001; Cramer V = 0.21). Perceptions about running long distances became more negative after reading the online resource, with more participants believing it could increase the risk of knee OA (65.2% vs 47.0%; χ^2^ [df = 3; n = 2488] = 213.62; *P*<.001; Cramer V = 0.21).

After reading the online resource, more participants perceived that it was appropriate for nonrunners with knee OA to start running (88.3%) or for runners with knee OA to keep running (92.4%) if they had no symptoms before or after running, in comparison with the preinfographics responses (65.5% and 76.6%, respectively; *P*<.001). Lower proportions of participants perceived that people with knee OA who maintained running could increase their risk of getting more knee pain (11.7% vs 33.2%; χ^2^ [df = 3; n = 2488] = 475.34; *P*<.001; Cramer V = 0.31) or needing joint replacement surgery (7.3% vs 23.0%; χ^2^ [df = 3; n = 2488] = 498.46; *P*<.001; Cramer V = 0.32). Detailed frequencies and proportions are provided in SUPPLEMENTAL TABLE S2.

**FIGURE 2:**
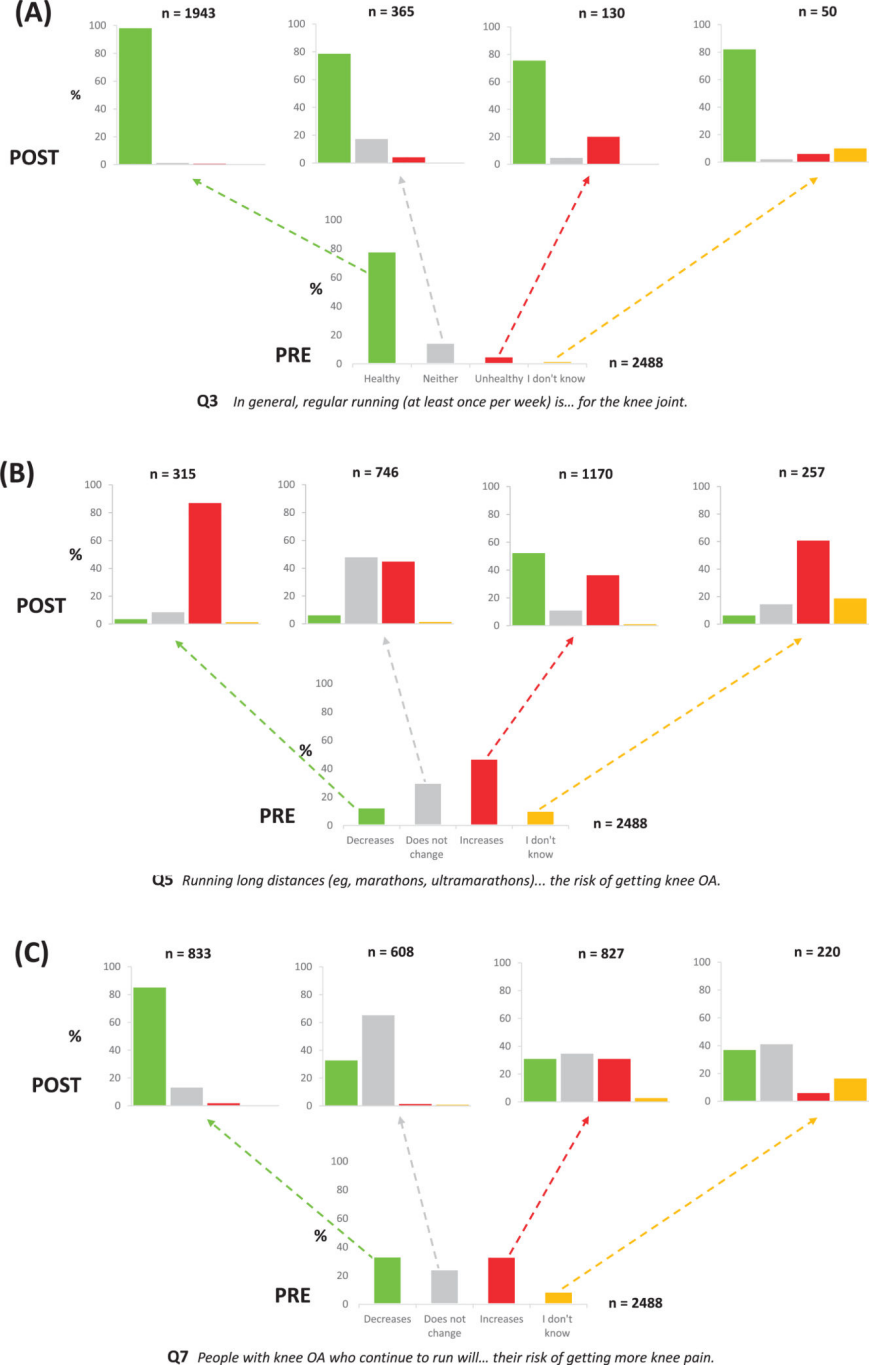
Proportions of posteducational resource responses based on pre-educational resource perceptions for questions Q3 (A), Q5 (B), and Q7 (C). Abbreviation: OA, osteoarthritis.

A breakdown of perception changes based on preinfographics responses to Q3, Q5, and Q7 is presented in FIGURE 2. Most participants who initially perceived regular running as unhealthy for the knee, neither healthy nor unhealthy for the knee, or were unsure, changed to more positive perceptions after reading the online resource (FIGURE 2A). Perceptions about running long distances became more negative (FIGURE 2B). Across all categories of responses, participants reported more positive or neutral perceptions about the risk of getting more knee pain in people with knee OA who maintained running (FIGURE 2C). Detailed comparisons of preinfographics and postinfographics responses (Q4, Q6, Q8, Q9) are presented in SUPPLEMENTAL FIGURE S3. Detailed frequencies and proportions of perception changes based on subgroups (runners, nonrunners, respondents with or without knee OA) are presented in SUPPLEMENTAL TABLE S4.

### Research Priorities

After viewing the online resource, participants identified the following topics as priorities for future research: identifying running parameters that minimize the risk of getting knee OA (69%), and recommendations about training modifications (63%) or running technique (57%) for runners with knee OA (TABLE 3). Three percent of participants responded that current research provided clear guidance on running and knee health.

## DISCUSSION

The freely available 7-language online education resource developed by our team was perceived by people as useful to inform the general public and health care professionals about running and knee health. Perceptions were generally more favorable to running after reading the infographics, with more participants perceiving running as beneficial for knee health in people with and without knee OA.

We provide a useful, scalable, and freely accessible resource. Education initiatives about knee health should be clear and comprehensive,^24^ and target common but erroneous perceptions that exercise and physical activity lead to joint pain and damage.^7^ People who believe that recreational running is harmful for their knees may avoid running altogether, even though recreational running could have positive effects on their knee health.^1^ Our online resource encouraged a positive participatory discourse^7,12^ and emphasized the beneficial effects of running on overall health and comorbidities in healthy people and in those with knee OA.^41^ After reading the material, people had more positive perceptions about running and knee health.

It remains unknown if the infographics used in this study can change behavior. Whether more positive perceptions would lead to greater running participation is not guaranteed, as other factors such as motivation and physical capacity play a large role in implementing a new behavior that is perceived as healthy.^21^ However, the usefulness and positive effects on perception of this series of infographics suggest it could complement other educational resources for individuals with knee OA^23,25^ and be used in future trials testing behavior change.

Respondents were less favorable to running long distances after viewing the infographics. Although we did not intend to imply a direct relationship between “running distance” and “risk of knee OA,” the concept of “ideal amount of running” proposed in the infographic likely had a different meaning for different people. The infographic was developed based on a systematic review showing a higher prevalence of knee OA in competitive runners (13%) than in recreational runners (3%) and sedentary people (10%).^1^ However, the review could not establish any association between knee OA and running distance. Even though our online resource was developed using feedback from consumers in terms of readability and comprehension of the content, this highlights the need to use knowledge translation principles and planned action theories to best translate the available evidence and influence change.^27^ This aspect should be clarified in future iterations of the online resource, which would also provide information on research priorities outlined by participants when it becomes available.

### Clinical Implications

Our open-access education resource is appropriate to use in large-scale initiatives to help individuals with and without knee OA make informed decisions, based on personal interest and current research, about the appropriateness of running. It could also be used in the clinical setting to educate patients about running and knee health.

### Limitations

Our education resource consisted of infographics. However, best practice about content and format for patient education varies between current guidelines for OA.^9^ These guidelines unfortunately do not always meet the patient’s education needs,^26^ and other formats such as videos or podcasts could have been more interesting for some participants. Because our online resource was targeted at individuals with and without knee OA, the evidence provided combined concepts that we felt were applicable to both populations. Patient-centered and targeted interventions to the user’s needs and behaviors are needed to best implement educational resources.

Even though we did not use a true codesign,^4^ we believe that significant feedback from knowledge users’ contributed to the perceived usefulness of the online resource. Our sample is relatively young (median 37 years) considering the high proportion of people with knee OA. Older individuals might have different perceptions regarding running and knee health. Two-thirds of our sample were runners, with likely different perceptions about the safety/suitability of running for knee health than nonrunners. In previous analyses of survey responses,^17^ perceptions were not strongly associated with running status. Additionally, changes in perception do not seem to differ between subgroup of runners vs nonrunners, or participants with and without knee OA.

We decided to collapse scales for data analysis (eg, somewhat appropriate with very appropriate). While this reduced the variability of our data, it helped to avoid problems linked with “empty categories” and facilitated the interpretation of our findings. From a practical perspective, moving someone’s perceptions from “somewhat inappropriate” to either “somewhat appropriate” or “very appropriate” after reading the resource should be considered positive, and an indication that the intervention did affect perceptions.

Our sample could have been more diverse. The impact of education resources on health outcomes may differ depending on the language, literacy level, and cultural background of the intended audience. Because of the 7 languages used in this study, our sample primarily included participants from Western cultures. Furthermore, the authors’ networks likely reached a sample mainly composed of highly educated individuals. As such, the results may not generalize to people from other parts of the world, who may have different beliefs and attitudes toward running and physical activity, and those with lower levels of education and socioeconomic status. However, several coauthors provide education on running to those in these networks. Thus, our finding that 76.8% changed their perceptions after the infographics likely represents the low end in the general population.

Education resources on their own may not necessarily lead to behavior change. Because of the nature of the study design (ie, testing perceptions immediately after reading the infographics), the long-term effects on perceptions about running and knee health remain unknown. We believe our findings provide a basis for educational interventions to change behavior and improve health outcomes in runners with and without knee OA. We also believe that future research priorities identified by participants will inspire future work.

## CONCLUSION

A free online evidence-based educational resource about running and knee health was useful for the public and health care professionals. After reading the material, most people had more positive perceptions about running and knee health.

## Supplementary Material

Educational Module

Supplemental Figure S1

Supplemental Figure S3

Supplemental Table S2

Supplemental Table S4

## Figures and Tables

**TABLE 1:** Posteducational Resource Questions (All Mandatory, Structured in 3 to 5 Pages Based on Adaptive Questioning)

Q1. How would you rate the usefulness of the educational module to learn about research on running and knee joint health?	Visual analog scale (slider bar with 1 decimal), from 0 (not useful) to 10 (very useful)
Q2. To what degree have your perceptions about running and knee health changed after the educational module?	• Not at all (skip to question 10) • A little bit (skip to question 10) • A moderate amount (answer all remaining questions) • A lot (answer all remaining questions)
Q3. In general, regular running (at least once per week) is ___________ for the knee joint.	• Very healthy • Somewhat healthy • Neither healthy nor unhealthy • Somewhat unhealthy • Very unhealthy • I don’t know
Q4. Running frequently (at least 3 times per week) ____________ the risk of getting knee OA.	• Greatly increases • Somewhat increases • Does not change • Somewhat decreases • Greatly decreases • I don’t know
Q5. Running long distances (such as marathons and ultramarathons) ____________ the risk of getting knee OA.	• Greatly increases • Somewhat increases • Does not change • Somewhat decreases • Greatly decreases • I don’t know
Q6. It is _________ for a nonrunner with knee OA to start a running program if they don’t have symptoms before or after they go running.	• Very appropriate • Somewhat appropriate • Neither appropriate or inappropriate • Somewhat inappropriate • Very inappropriate • I don’t know
Q7. People with knee OA who continue to run will __________ their risk of getting more knee pain.	• Greatly increase • Somewhat increase • Not change • Somewhat decrease • Greatly decrease • I don’t know
Q8. People with knee OA who keep running regularly will ____________ the need for joint replacement surgery.	• Greatly increase • Somewhat increase • Not change • Somewhat decrease • Greatly decrease • I don’t know
Q9. It is _________ for runners who have knee OA to continue if they don’t have symptoms before or after they go running.	• Very appropriate • Somewhat appropriate • Neither appropriate or inappropriate • Somewhat inappropriate • Very inappropriate • I don’t know
Q10. What questions still need to be answered for you to provide better guidance about running and knee health? (Please select all that apply.)	• Identifying running parameters that minimize the risk of getting knee OA (running distance, speed, frequency, level of competition, etc) level of competition, etc) • If running with knee OA will create damage to structures of the knee • If running with knee OA will aggravate the severity of knee pain • Recommendations about training modifications for runners with knee OA (stop vs continue, modify distance, speed, frequency, etc) • The usefulness of changing running technique in runners with knee OA • The usefulness of changing running surface in runners with knee OA • The usefulness of changing running shoes in runners with knee OA • Safety of running after a knee replacement surgery • Other (please specify) • No more research is needed; clear guidance and recommendations about running and knee health can be provided based on existing research

Abbreviation: OA, osteoarthritis.

**TABLE 2:** Demographics of Participants

	General Public (n = 1291)	Health Care Professionals (n = 1403)	Total (n = 2694)

**Age (years, median [IQR]) Gender, n (%)**	41 (30-50)	33 (28-43)	37 (29-47)
Woman	668 (51.7)	679 (48.4)	1347 (50)
Man	614 (47.6)	722 (51.5)	1336 (46.6)
Gender-fluid	2 (0.2)	0 (0)	2 (0.1)
Nonbinary	3 (0.2)	0 (0)	3 (0.1)
Two-spirit	1 (0.1)	1 (0.1)	2 (0.1)
Prefer not to answer	3 (0.2)	1 (0.1)	4 (0.1)
**Level of education, n (%)**			
Below high school	16 (1.2)	0 (0)	16 (0.6)
High school	108 (8.4)	11 (0.8)	119 (4.4)
Nonuniversity	174 (13.5)	35 (2.5)	209 (7.8)
University	993 (76.9)	1357 (96.7)	2350 (87.2)
**General health status, n (%)**			
Excellent	378 (29.3)	511 (36.4)	889 (33)
Very good	627 (48.6)	674 (48)	1301 (48.3)
Good	252 (19.5)	198 (14.1)	450 (16.7)
Fair	32 (2.5)	18 (1.3)	50 (1.9)
Poor	2 (0.2)	2 (0.1)	4 (0.1)
**Diagnosed with knee OA, n(%)** **History of knee injury, n(%)**	1094 (84.7)	136 (9.7)	1230 (45.7)
No	471 (36.5)	516 (36.8)	987 (36.6)
Yes, without surgery	628 (48.6)	685 (48.8)	1313 (48.7)
Yes, with surgery	192 (14.9)	202 (14.4)	394 (14.6)
**Self-identified runner n (%)** **Profession, n (%)**	1087 (84.2)	733 (52.2)	1820 (67.6)
Medical doctor		83 (5.9)	
Physiotherapist		1132 (80.7)	
Chiropractor		66 (4.7)	
Athletic therapist/trainer		78 (5.6)	
Podiatrist/pedorthist		13 (0.9)	
Osteopath		26 (1.9)	
Nurse		20 (1.4)	
Other		84 (6.0)	

**TABLE 3:** Research Priorities Identified by Participants (Unlimited Selections) (n = 2488)

	n	%

1. Identifying running parameters that minimize the risk of getting knee OA (running distance, speed, frequency, level of competition, etc)	1705	69
2. Recommendations about training modifications for runners with knee OA (stop vs continue, modify distance, speed, frequency, etc)	1556	63
3. The usefulness of changing running technique in runners with knee OA	1415	57
4. The usefulness of changing running surface in runners with knee OA	1171	47
5. The usefulness of changing running shoes in runners with knee OA	906	36
6. If running with knee OA will aggravate the severity of knee pain	496	20
7. If running with knee OA will create damage to structures of the knee	457	18
8. Safety of running after a knee replacement surgery	109	4
No more research is needed; clear guidance and recommendations about running and knee health can be provided based on existing research	63	3

## Data Availability

Survey data (premodule and postmodule) are available upon reasonable request. The educational module is available for free, in all 7 languages, in a public open-access repository (http://hdl.handle.net/2429/82767). Any further requests can be made to the corresponding author.

## KEY POINTS

### FINDINGS:

The free evidence-based online education resource about running and knee health developed in this study, and available in 7 languages, was considered useful by the public and health care professionals. After reading the module, respondents had more positive perceptions about regular running, frequent running, and running with knee osteoarthritis. Running long distances was perceived more negatively.

### IMPLICATIONS:

The proposed educational resource can be presented to the general population, patients, and health care professionals to effectively change perceptions about running and knee health.

### CAUTION:

Further research is needed to explore the effects of this educational resource on behavior change and running participation.
