# Supplementary material for: An Online Evidence-Based Education Resource Is Useful and Can Change People’s Perceptions About Running and Knee Health
Source: JOSPT Open. Author manuscript; Available in PMC 2025 Apr 3. (PMC11967912; doi:10.2519/josptopen.2024.0149)
Supplement: Educational Module [file NIHMS2048133-supplement-Educational_Module.pdf]

Running and knee health

Please note that this online educational module is intended to provide a brief overview of the research about running and knee health. Detailed references are provided at the end of the module.

This module is not meant to provide a treatment plan. If you have knee pain, consult a qualified healthcare professional.

We acknowledge and appreciate the contribution of multiple runners, non-runners and healthcare providers who helped in the development of this educational module.

- The authors

Jean-Francois Esculier PT, PhD (University of British Columbia, Canada)
Manuela Besomi PT, MSc (University of Queensland, Australia)
Danilo De Oliveira Silva PT, PhD (La Trobe University, Australia)
Anthony Teoli PT, MSc (McGill University, Canada)
Christian Barton PT, PhD (La Trobe University, Australia)
Bill Vicenzino PT, PhD (University of Queensland, Australia)
Marienke van Middelkoop PT, PhD (Erasmus Medical Centre, The Netherlands)
Rich Willy PT, PhD (University of Montana, USA)
Matthew Harkey AT, PhD (Tufts Medical Centre, USA)
Michael Callaghan PT, PhD (Manchester Metropolitan University, United Kingdom)
Michael Rathleff PT, PhD (Aalborg University, Denmark)
Alison Hoens PT, MSc (University of British Columbia, Canada)
Michael A. Hunt PT, PhD (University of British Columbia, Canada)

Project lead

UBC

Collaborators

The University of Queensland Australia — Create Change

La Trobe University

McGill University

Erasmus MC University Medical Center Rotterdam

University of Montana

Manchester Metropolitan University

Tufts Medical Center

Aalborg University Denmark

What is Knee osteoarthritis (OA)?

The **most common** joint disease and a major cause of **disability and pain.** It affects bone, cartilage, ligaments and muscles.

Osteoarthritis (OA) currently affects **240 million** people globally.

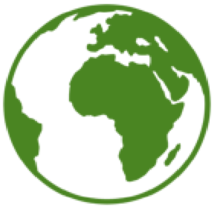

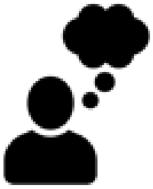

How does knee OA impact life?

Physical and psychosocial impact

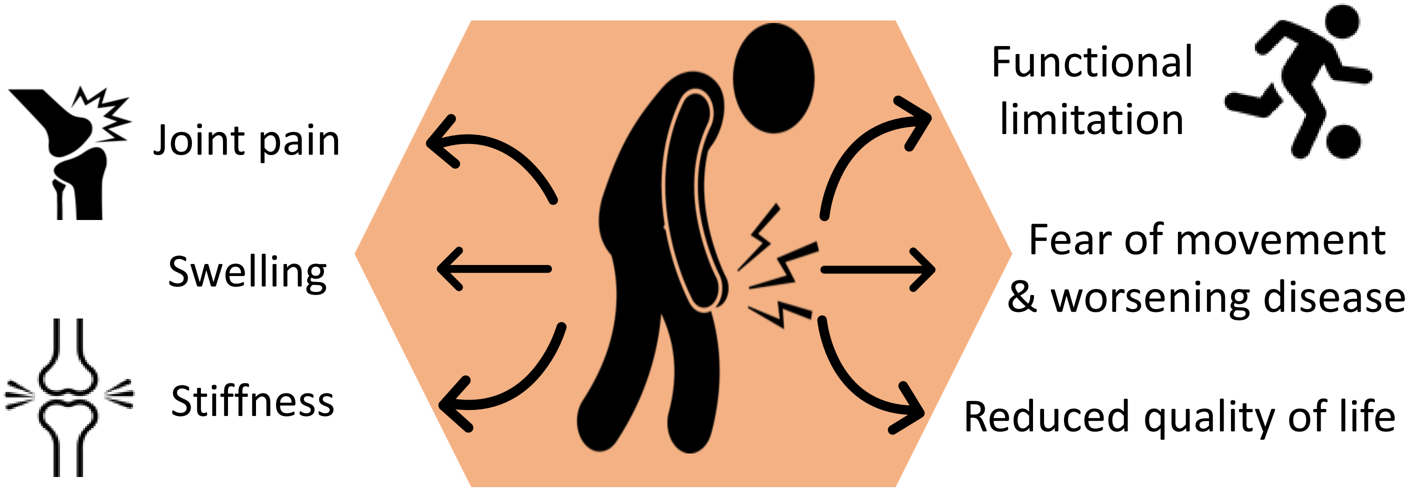

References

Osteoarthritis Research Society International (2019), Wallis et al. (2019)

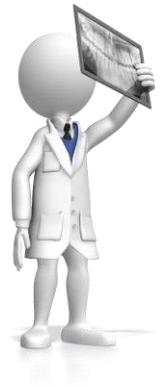

SHOULD I BE CONCERNED ABOUT THE RESULTS OF MY MEDICAL IMAGING EXAMS?

Findings from medical imaging are **not necessarily related** with pain. As many as 14% of adults aged less than 40 years and 43% of adults over 40 years have OA on imaging, but no pain!

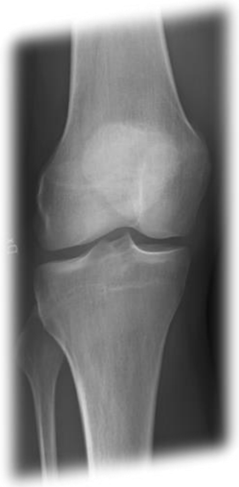

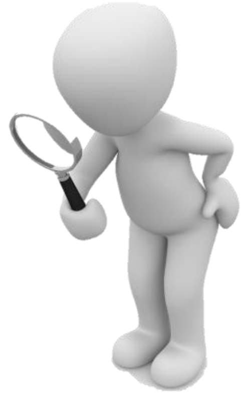

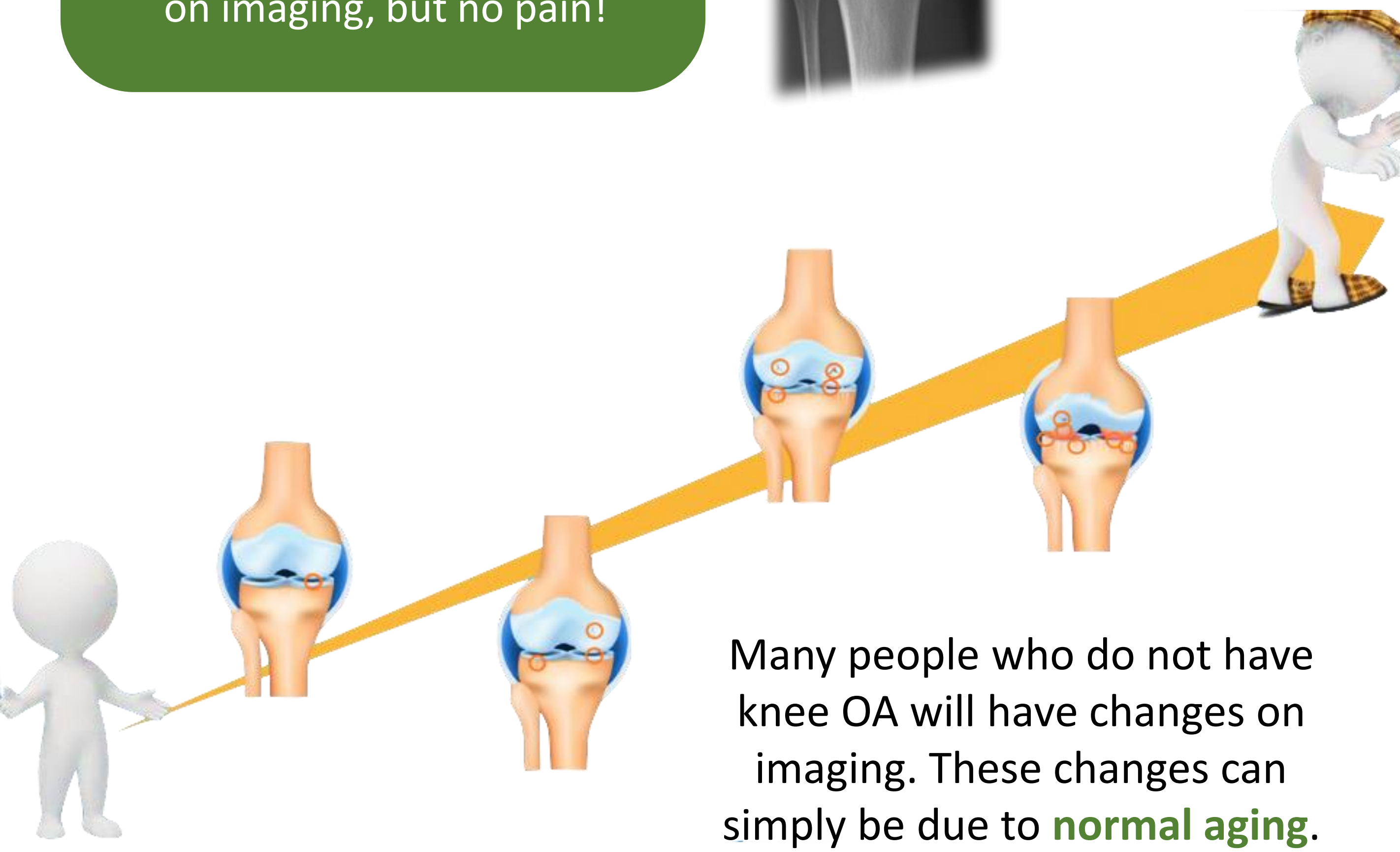

Many people who do not have knee OA will have changes on imaging. These changes can simply be due to **normal aging**.

References

Culvenor et al. (2019), Finan (2013)

PEOPLE ARE MORE LIKELY TO GET KNEE OA IF THEY...

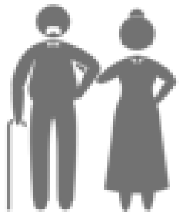

Are 50 years or older

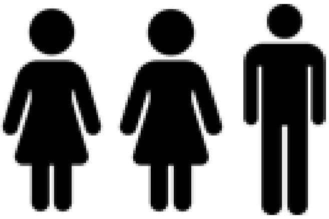

Are female

2x more likely than males

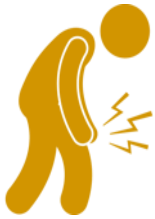

Have had a serious knee injury

3x more likely

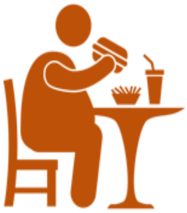

Are overweight or obese

3x more likely

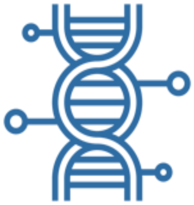

Have a family history of knee OA

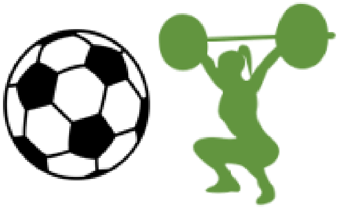

Have played high-level sports

References

Driban et al. (2017), Silverwood et al. (2015)

UBC

RUNNING IS GOOD FOR OVERALL HEALTH!

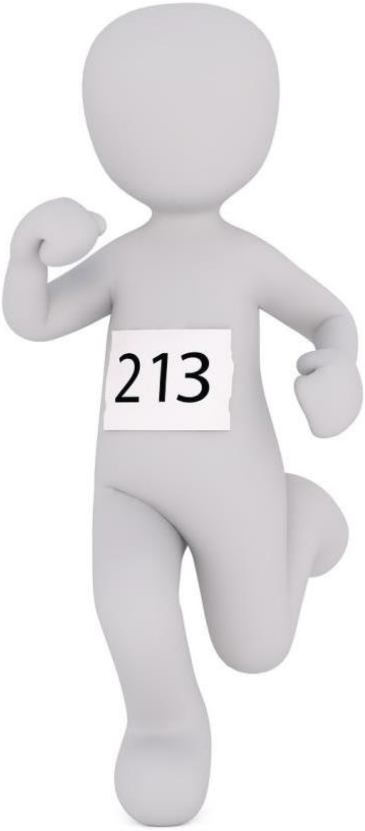

Mortality rate due to cardiovascular disease, cancer or infection

Mental health: improved sleep, symptoms of depression, psychological functioning, etc.

Ability to control **weight**

Better for knee joint health

Aerobic **fitness** & heart and lung **capacity**

Physical abilities

References

Pedisic et al. (2019), Eime et al. (2013), Hespanhol et al. (2015), Kalak et al. (2012), Szabo & Ábrahám (2013), Oja et al. (2015)

Does running cause knee OA?

NOT NECESSARILY!

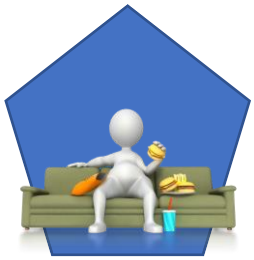

10 out of 100
sedentary people (non-runners) have knee OA

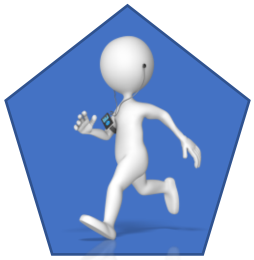

3 out of 100
recreational runners have knee OA

**Recreational means Non-professional or Amateur levels*

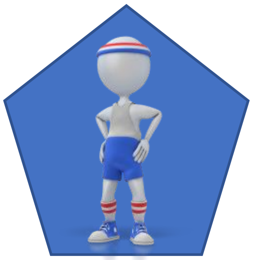

13 out of 100
elite runners have knee OA

**Elite means Professional or International levels*

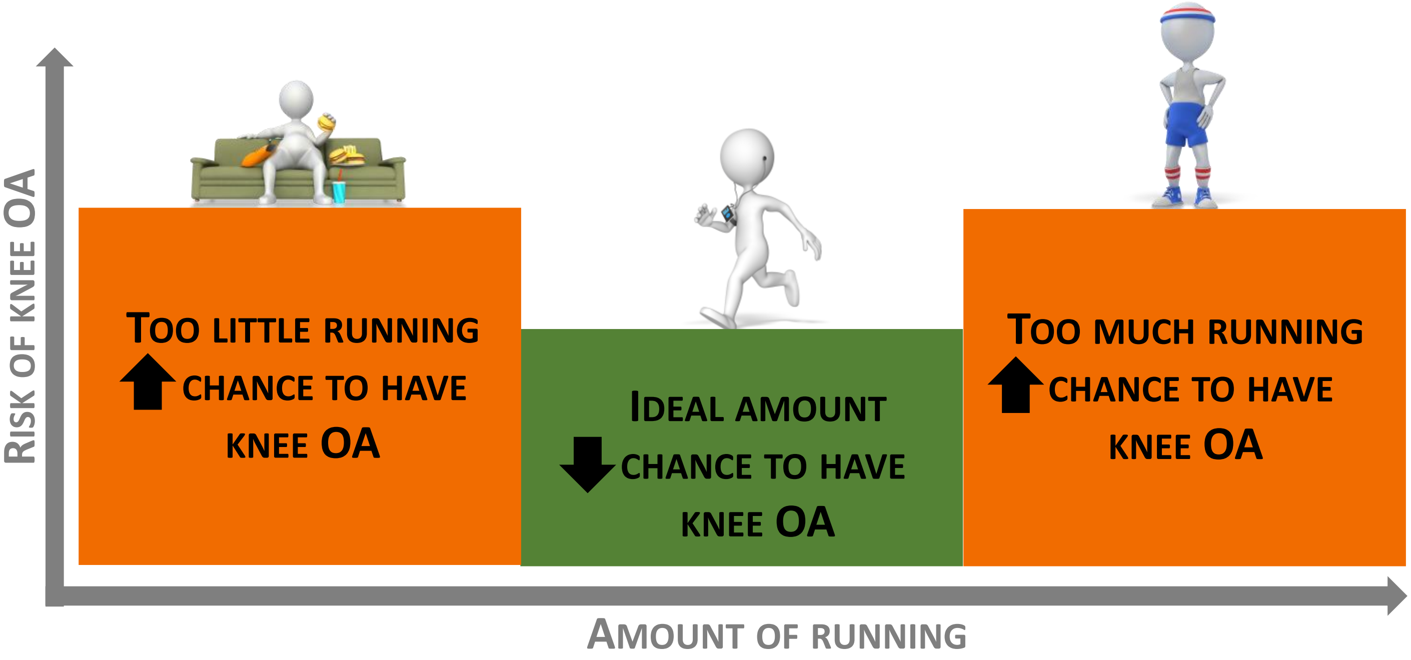

DID YOU KNOW?

Running may have a **protective effect.** Runners seem to have a **54% lower** chance for needing knee surgery due to OA (knee replacement).

References
Alentorn-Geli et al. (2017), Timmins et al. (2017)

What if running helped your knees get stronger?

Running can have a **positive effect** on the **knee** when the **ideal load** (dose of activity) is applied

Increased **bone** density (solid bones)

Increased capacity of **muscles**

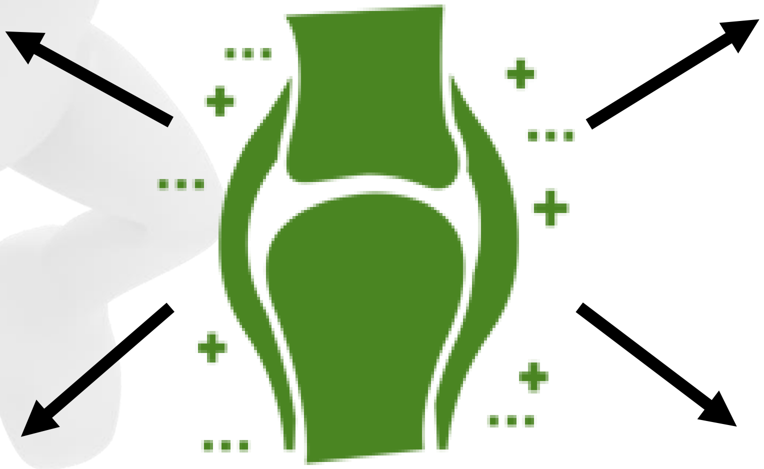

Less **inflammation** in the healthy knee

More robust **cartilage**

Healthy bones, muscles and cartilage can help maintain healthy knees! Note that the **ideal load varies** according to many factors (age, habits, sleep, etc.) and is **different from one person to the other**.

UBC

References

Horga et al. (2019), Hyldahl et al. (2016), Van Ginckel et al. (2010)

IS RUNNING BAD FOR PEOPLE WITH KNEE OA?

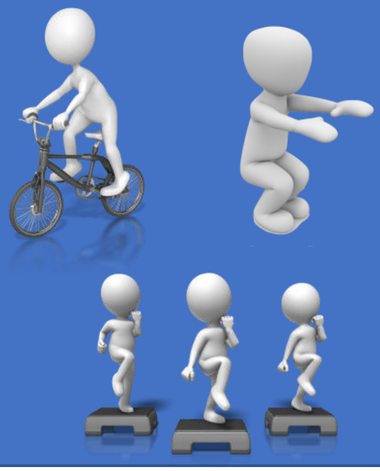

Exercise is **helpful** for people with knee OA.

Exercising with **moderate impact** is **not harmful** for the knee cartilage.

DID YOU KNOW?

?

Recreational running in people with knee OA...

- Does NOT seem to increase symptoms
- Does NOT seem to damage knee joint structures
- May require more recovery time following a run

Runners with knee OA may wish to consider adjusting training according to symptoms. Example: run shorter distances, run a little bit slower, avoid downhill running.

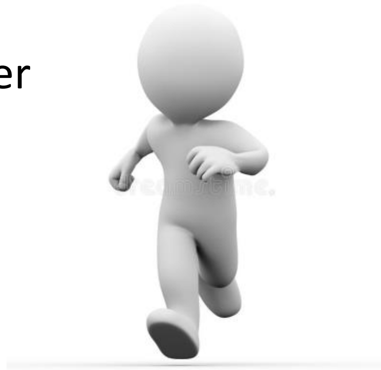

References

Bricca et al. (2019), Esculier et al. (2019), Lo et al. (2018)

IN SUMMARY, RUNNING MAY BE GOOD FOR KNEE HEALTH!

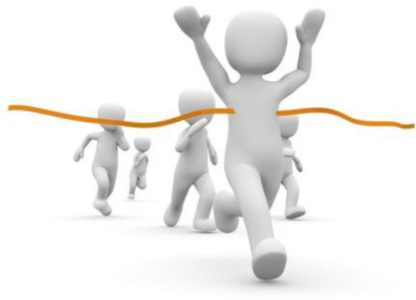

1. Knee OA is a **common** cause of pain and disability

2. **Running** is great for **physical** health, **mental** health and **knee** health

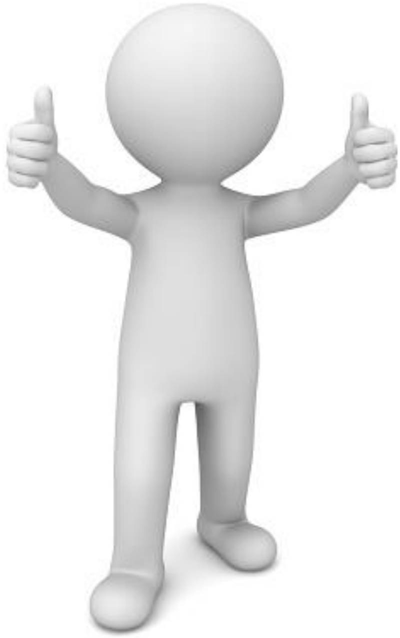

3. Knee OA is **less frequent** in **recreational runners** than in sedentary people or elite runners

4. Recreational running may have a **positive effect** on knee health by making **bones**, **cartilage** and **muscles** stronger

5. **Exercise** is **good** for people with knee OA. Running provides a simple inexpensive option to maintain good exercise habits

**More studies are needed to provide specific guidelines on optimal running parameters*

UBC

References

1. **Alentorn-Geli E. et al. (2017).** The association of recreational and competitive running with hip and knee osteoarthritis: a systematic review and meta-analysis. Journal of Orthopaedic & Sports Physical Therapy 47(6):373-390.
2. **Bricca A. et al. (2019).** Impact of exercise on articular cartilage in people at risk of, or with established, knee osteoarthritis: a systematic review of randomised controlled trials. British Journal of Sports Medicine 53(15):940-947.
3. **Culvenor A.G. et al. (2019).** Prevalence of knee osteoarthritis features on magnetic resonance imaging in asymptomatic uninjured adults: a systematic review and meta-analysis. British Journal of Sports Medicine 53(20):1268-1278.
4. **Driban J.B. et al. (2017).** Is participation in certain sports associated with knee osteoarthritis? A systematic review. Journal of Athletic Training 52(6):497-506.
5. **Eime R.M. et al. (2013).** A systematic review of the psychological and social benefits of participation in sport for adults. International Journal of Behavioural Nutrition and Physical Activity 10:135.
6. **Esculier J.F. et al. (2019).** Cartilage recovery in runners with and without knee osteoarthritis: a pilot study. The Knee (In press).
7. **Finan P.H. (2013).** Discordance between pain and radiographic severity in knee osteoarthritis: Findings from quantitative sensory testing of central sensitization. Arthritis & Rheumatism 65(2):363–372.
8. **Hespanhol Junior L.C. et al. (2015).** Meta-analyses of the effects of habitual running on indices of health in physically inactive adults. Sports Medicine 45(10):1455-1468.
9. **Horga L.M. et al. (2019).** Can marathon running improve knee damage of middle-aged adults? A prospective cohort study. BMJ Open Sport & Exercise Medicine 5: e000586
10. **Hyldahl R.D. et al. (2016).** Running decreases knee intra-articular cytokine and cartilage oligomeric matrix concentrations: a pilot study. European Journal of Applied Physiology 116(11-12):2305-2314.
11. **Kalak N. et al. (2012).** Daily morning running for 3 weeks improved sleep and psychological functioning in healthy adolescents compared with controls. Journal of Adolescent Health 51(6):615-622.
12. **Lo G.H. et al. (2018).** Running does not increase symptoms or structural progression in people with knee osteoarthritis: data from the osteoarthritis initiative. Clinical Rheumatology 37(9):2497-2504.
13. **Oja P. et al. (2015).** Health benefits of different sport disciplines for adults: systematic review of observational and intervention studies with meta-analysis. British Journal of Sports Medicine 49(7):434-440.
14. **Osteoarthritis Research Society International (OARSI) 2019**: http://www.oarsi.org
15. **Pedisic Z. et al. (2019).** Is running associated with a lower risk of all-cause, cardiovascular and cancer mortality, and is the more the better? A systematic review and meta-analysis. British Journal of Sports Medicine (early online)
16. **Silverwood V. et al. (2015).** Current evidence on risk factors for knee osteoarthritis in older adults: a systematic review and meta-analysis. Osteoarthritis & Cartilage 23(4):507-515.
17. **Szabo A. & Ábrahám J. (2013).** The psychological benefits of recreational running: a field study. Psychology, Health & Medicine 18(3):251-261.
18. **Timmins K.A. et al. (2017).** Running and knee osteoarthritis: a systematic review and meta-analysis. American Journal of Sports Medicine 45(6):1447-1457.
19. **Van Ginckel A. et al. (2011).** Functional adaptation of knee cartilage in asymptomatic female novice runners compared to sedentary controls. A longitudinal analysis using delayed Gadolinium Enhanced Magnetic Resonance Imaging of Cartilage (dGEMRIC). Osteoarthritis & Cartilage 18(12):1564-1569.
20. **Wallis J.A. et al. (2019).** Experience of living with knee osteoarthritis: a systematic review of qualitative studies. BMJ Open 9(9):e030060.
