# Supplementary material for: An Online Evidence-Based Education Resource Is Useful and Can Change People’s Perceptions About Running and Knee Health
Source: JOSPT Open. Author manuscript; Available in PMC 2025 Apr 3. (PMC11967912; doi:10.2519/josptopen.2024.0149)
Supplement: Supplemental Figure S1 [file NIHMS2048133-supplement-Supplemental_Figure_S1.docx]

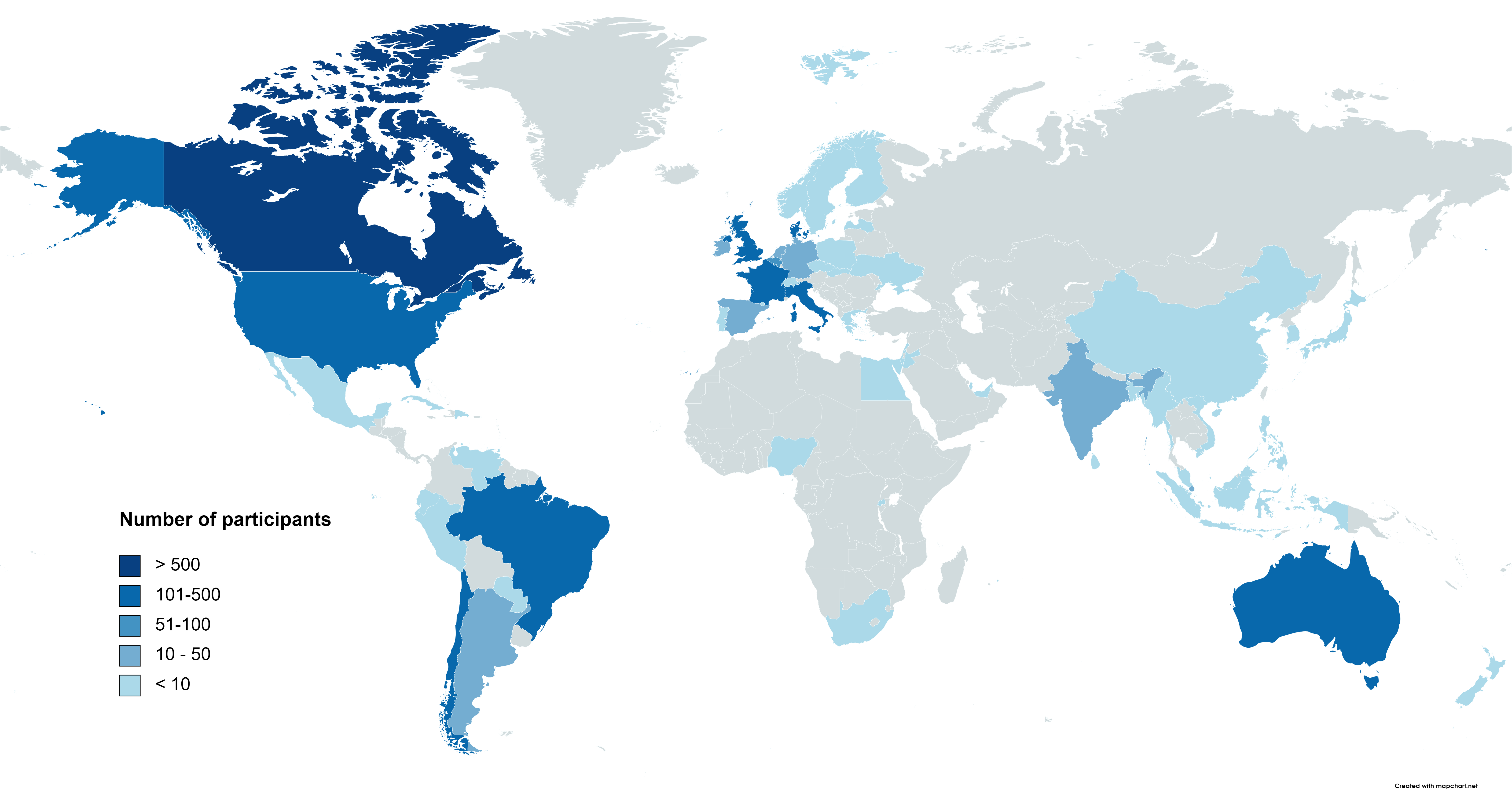


**SUPPLEMENTARY FIGURE S1.** Geographical distribution of participants (map created with mapchart.net)
