# Supplementary material for: An Online Evidence-Based Education Resource Is Useful and Can Change People’s Perceptions About Running and Knee Health
Source: JOSPT Open. Author manuscript; Available in PMC 2025 Apr 3. (PMC11967912; doi:10.2519/josptopen.2024.0149)
Supplement: Supplemental Figure S3 [file NIHMS2048133-supplement-Supplemental_Figure_S3.pdf]

(A)

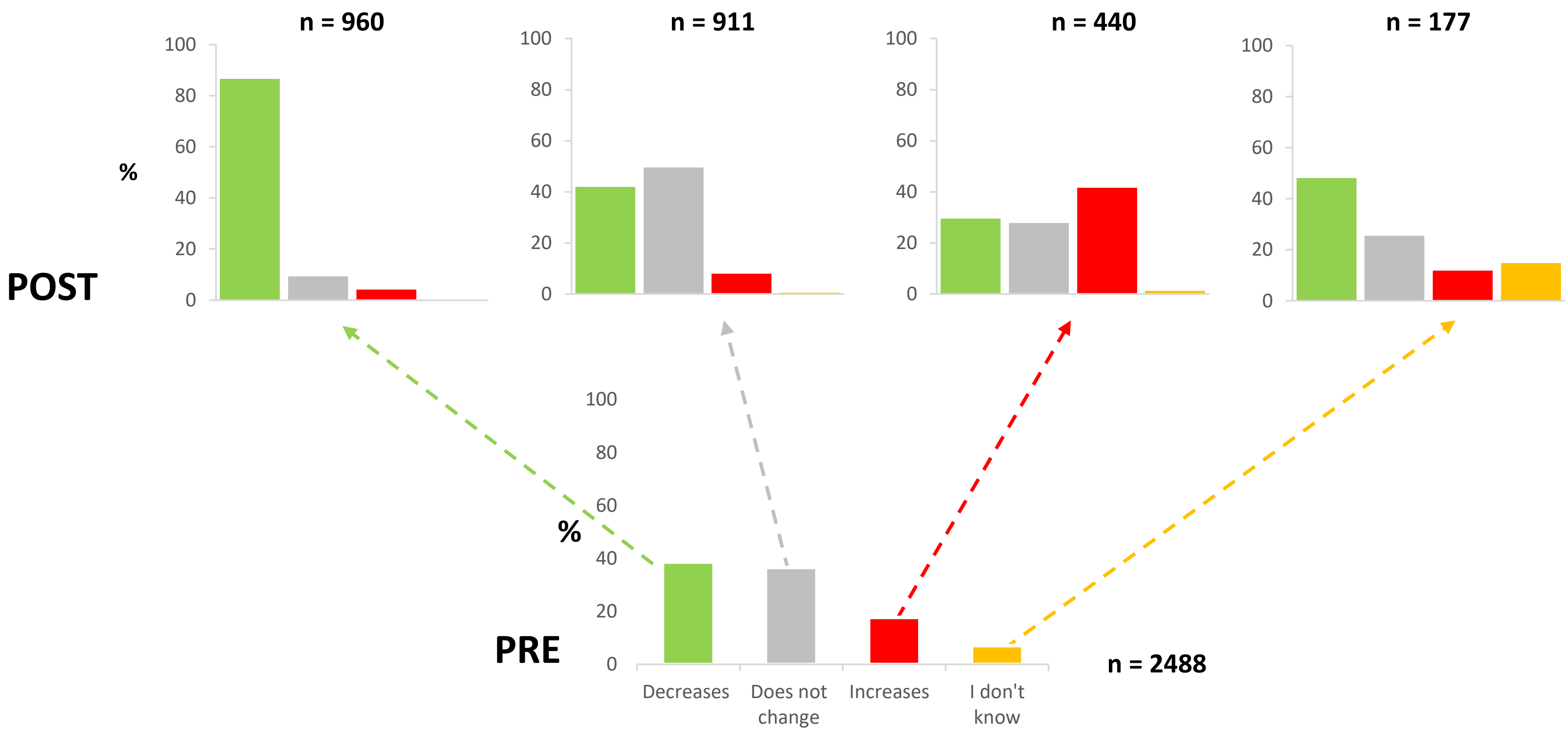

Q4 *Running frequently (at least 3 times per week)… the risk of getting knee OA.*

(B)

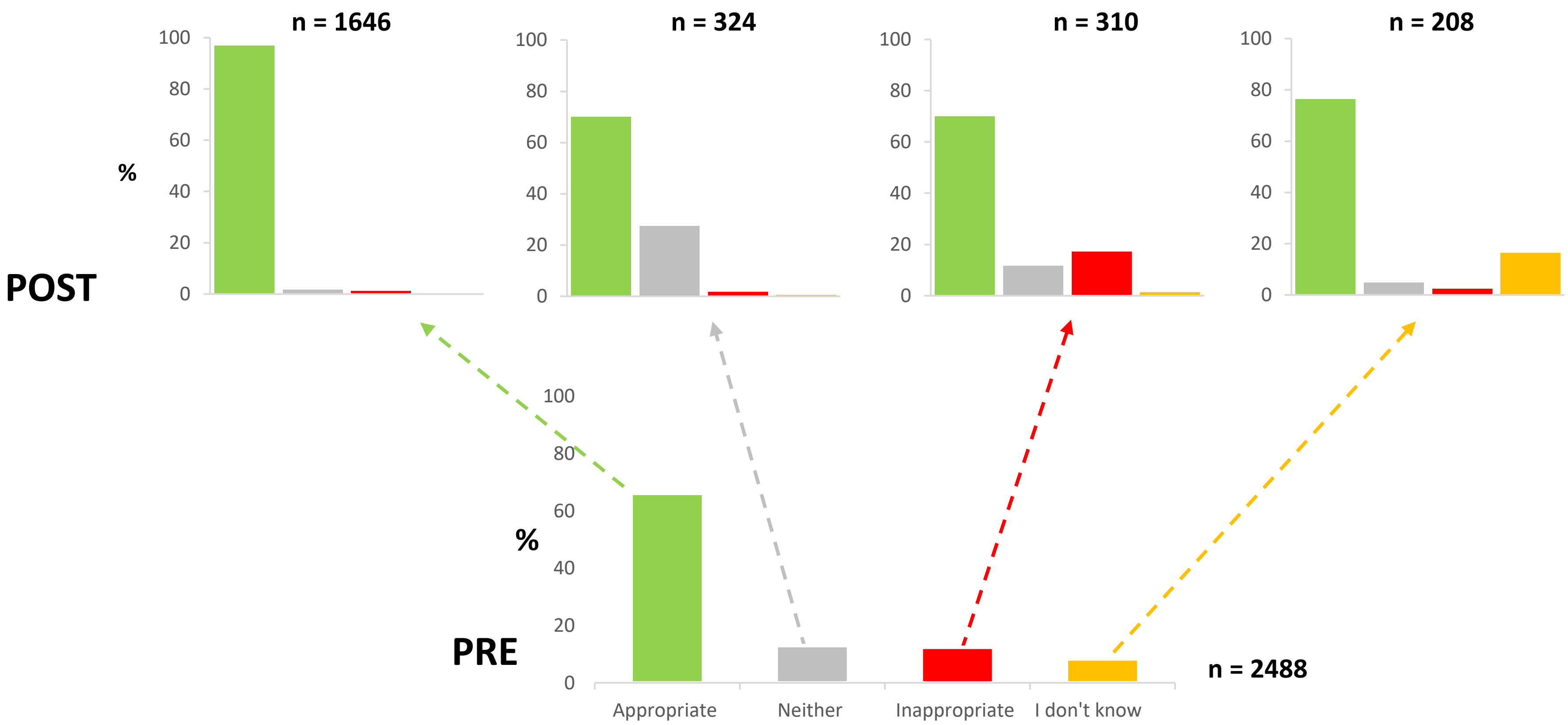

Q6 *It is… for a non-runner with knee OA to start a running program if they don't have symptoms before or after they go running.*

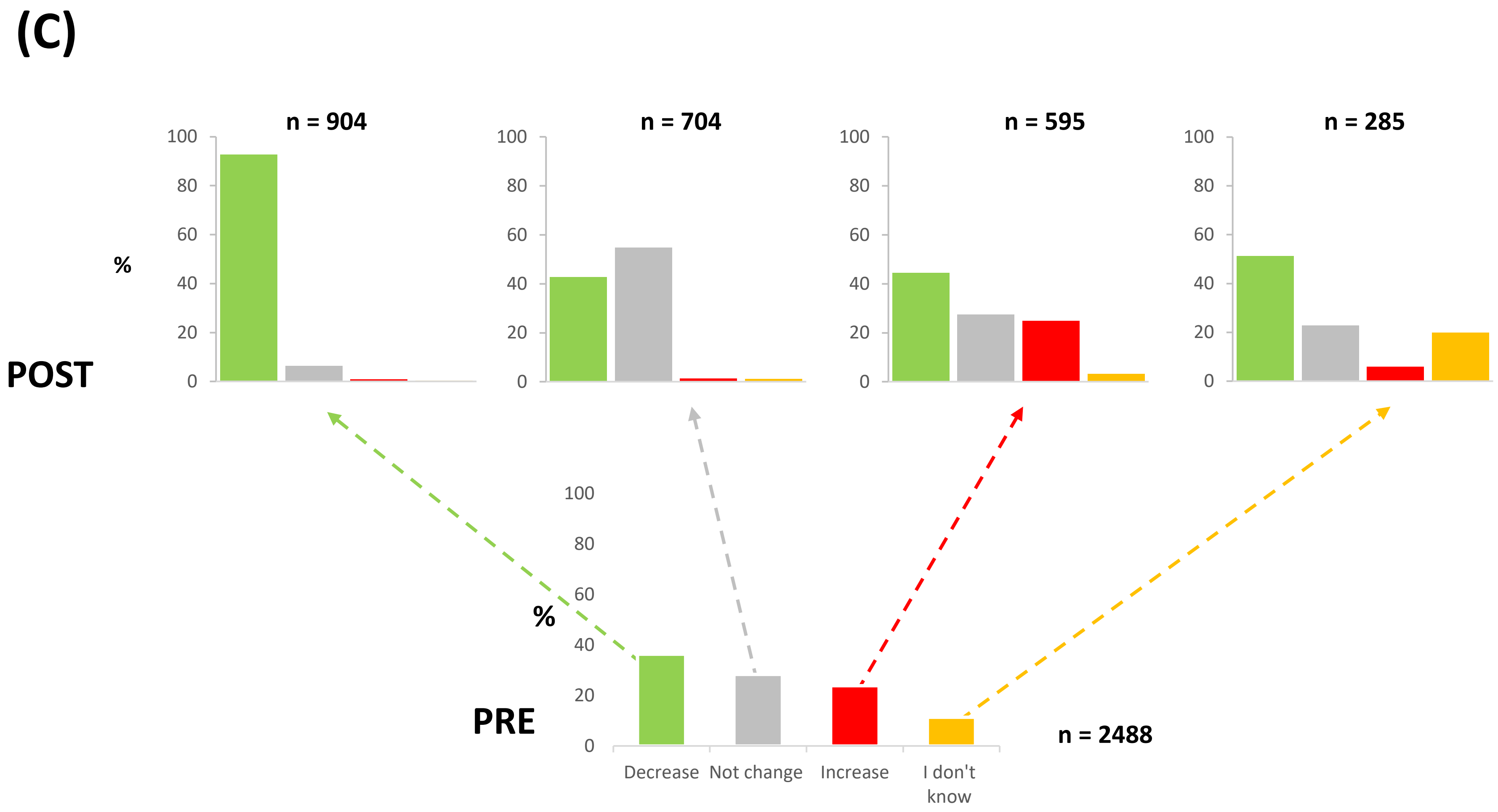

Q8 *People with knee OA who keep running regularly will… the need for joint replacement surgery*

(D)

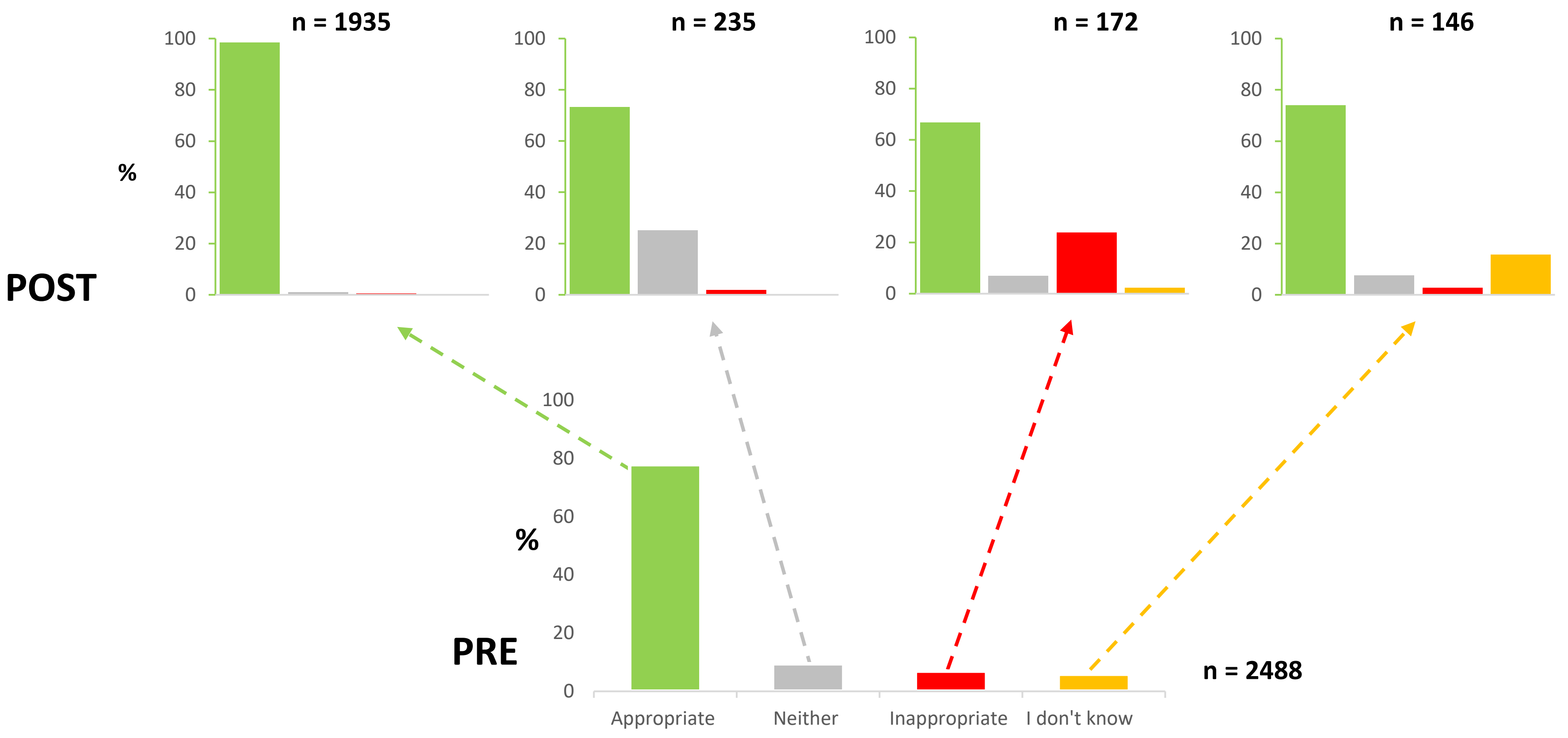

Q9 *It is… for runners who have knee OA to continue if they don't have symptoms before or after they go running.*

SUPPLEMENTARY FIGURE S3. Proportions of post-educational resource responses based on pre-educational resource perceptions about running for questions (A) Q4, (B) Q6, (C) Q8 and (D) Q9. OA = osteoarthritis.
