# Supplementary material for: An Online Evidence-Based Education Resource Is Useful and Can Change People’s Perceptions About Running and Knee Health
Source: JOSPT Open. Author manuscript; Available in PMC 2025 Apr 3. (PMC11967912; doi:10.2519/josptopen.2024.0149)
Supplement: Supplemental Table S2 [file NIHMS2048133-supplement-Supplemental_Table_S2.docx]

| **Supplementary Table S2. Detailed response proportions for pre- and post-module questions (n=2488).** | | | |
| --- | --- | --- | --- |
|  |  |  |  |
|  | **PRE** | **POST** | ***P*-value** |
|  |  |  |  |
| **Q3. In general, regular running (at least once per week) is ___________for the knee joint.** | | | |
| **Healthy (very, somewhat)** | 78.1% | 93.7% | **<0.001** |
| **Neither healthy nor unhealthy** | 14.7% | 3.7% | **<0.001** |
| **Unhealthy (somewhat, very)** | 5.2% | 2.3% | **<0.001** |
| **I don’t know** | 2.0% | 0.3% | **<0.001** |
|  | χ2 (3, n=2488) = 258.32  Cramer V = 0.228  **(moderate effect size)** | | **<0.001** |
|  |  |  |  |
| **Q4. Running frequently (at least 3 times per week) ____________ the risk of getting knee OA.** | | | |
| **Increases (greatly, somewhat)** | 17.7% | 12.7% | **<0.001** |
| **Does not change** | 36.6% | 28.4% | **<0.001** |
| **Decreases (somewhat, greatly)** | 38.6% | 57.4% | **<0.001** |
| **I don’t know** | 7.1% | 1.5% | **<0.001** |
|  | χ2 (3, n=2488) = 227.91  Cramer V = 0.214  **(moderate effect size)** | | **<0.001** |
|  |  |  |  |
| **Q5. Running long distances (such as marathons and ultra-marathons) ____________ the risk of getting knee OA.** | | | |
| **Increases (greatly, somewhat)** | 47.0% | 65.2% | **<0.001** |
| **Does not change** | 30.0% | 21.1% | **<0.001** |
| **Decreases (somewhat, greatly)** | 12.7% | 10.7% | 0.014 |
| **I don’t know** | 10.3% | 3.1% | **<0.001** |
|  | χ2 (3, n=2488) = 213.62  Cramer V = 0.207  **(moderate effect size)** | | **<0.001** |
|  |  |  |  |
| **Q6. It is _________ for a non-runner with knee OA to start a running program if they don’t have symptoms before or after they go running.** | | | |
| **Appropriate (very, somewhat)** | 66.2% | 88.3% | **<0.001** |
| **Neither appropriate nor inappropriate** | 13.0% | 6.6% | **<0.001** |
| **Inappropriate (somewhat, very)** | 12.5% | 3.3% | **<0.001** |
| **I don’t know** | 8.4% | 1.8% | **<0.001** |
|  | χ2 (3, n=2488) = 370.34  Cramer V = 0.273  **(moderate effect size)** | | **<0.001** |
|  |  |  |  |
| **Q7. People with knee OA who continue to run will __________ their risk of getting more knee pain.** | | | |
| **Increase (greatly, somewhat)** | 33.2% | 11.7% | **<0.001** |
| **Not change** | 24.4% | 35.5% | **<0.001** |
| **Decrease (somewhat, greatly)** | 33.5% | 50.2% | **<0.001** |
| **I don’t know** | 8.8% | 2.6% | **<0.001** |
|  | χ2 (3, n=2488) = 475.34  Cramer V = 0.309  **(large effect size)** | | **<0.001** |
|  |  |  |  |
| **Q8. People with knee OA who keep running regularly will ____________ the need for joint replacement surgery.** | | | |
| **Increase (greatly, somewhat)** | 23.9% | 7.3% | **<0.001** |
| **Not change** | 28.3% | 27% | 0.1478 |
| **Decrease (somewhat, greatly)** | 36.3% | 62.3% | **<0.001** |
| **I don’t know** | 11.5% | 3.5% | **<0.001** |
|  | χ2 (3, n=2488) = 498.46  Cramer V = 0.317  **(large effect size)** | | **<0.001** |
|  |  |  |  |
| **Q9. It is _________ for runners who have knee OA to continue if they don’t have symptoms before or after they go running.** | | | |
| **Appropriate (very, somewhat)** | 77.8% | 92.4% | **<0.001** |
| **Neither appropriate nor inappropriate** | 9.5% | 4.1% | **<0.001** |
| **Inappropriate (somewhat, very)** | 6.9% | 2.5% | **<0.001** |
| **I don’t know** | 5.9% | 1.1% | **<0.001** |
|  | χ2 (3, n=2488) = 217.47  Cramer V = 0.209  **(moderate effect size)** | | **<0.001** |
|  |  |  |  |
