# Supplementary material for: An Online Evidence-Based Education Resource Is Useful and Can Change People’s Perceptions About Running and Knee Health
Source: JOSPT Open. Author manuscript; Available in PMC 2025 Apr 3. (PMC11967912; doi:10.2519/josptopen.2024.0149)
Supplement: Supplemental Table S4 [file NIHMS2048133-supplement-Supplemental_Table_S4.docx]

**Supplementary Table S4**. Number and proportions of respondents who switched to more favourable or unfavourable perceptions after viewing the online resource. Data is presented as N (%) based on subgroups of Runners (n=1668), Non-Runners (n=820), Knee osteoarthritis (OA) (n=1120), Non knee OA (n=1360).

**Q3. In general, regular running (at least once per week) is ___________for the knee joint.**

*Healthy (very, somewhat)*

*Neither healthy nor unhealthy*

*Unhealthy (somewhat, very)*

*I don’t know*

| **Favourable changes after the online resource** | **Runners** | **Non-Runners** | **Knee OA** | **Non**  **knee OA** |
| --- | --- | --- | --- | --- |
| Unfavourable before, who then became favourable after | 70 (4.2) | 28 (3.4) | 63 (5.6) | 35 (2.6) |
| Unfavourable before, who then became neutral after | 2 (0.1) | 4 (0.5) | 1 (0.1) | 5 (0.4) |
| Neutral before, who then became favourable after | 186 (11.2) | 101 (12.3) | 172 (15.4) | 115 (8.4) |
| "I don't know" before, who became favourable after | 21 (1.3) | 20 (2.4) | 25 (2.2) | 16 (1.2) |

| **Unfavourable changes after the online resource** | **Runners** | **Non-Runners** | **Knee OA** | **Non**  **knee OA** |
| --- | --- | --- | --- | --- |
| Favourable before, who then became unfavourable after | 6 (0.4) | 8 (1) | 4 (0.4) | 10 (0.7) |
| Favourable before, who then became neutral after | 12 (0.7) | 10 (1.2) | 9 (0.8) | 13 (1) |
| Neutral before, who then became unfavourable after | 5 (0.3) | 10 (1.2) | 8 (0.7) | 7 (0.5) |
| "I don't know" before, who became unfavourable after | 2 (0.1) | 1 (0.1) | 3 (0.3) | 0 (0) |

|  | **Runners** | **Non-Runners** | **Knee OA** | **Non**  **knee OA** |
| --- | --- | --- | --- | --- |
| Did not change after the online resource | 1363 (74.9) | 636 (72.8) | 834 (67.8) | 1165 (79.6) |
| Changed to “I don’t know” after | 1 (0.1) | 1 (0.1) | 1 (0.1) | 1 (0.1) |

**Q4. Running frequently (at least 3 times per week) ____________ the risk of getting knee OA.**

*Increases (greatly, somewhat)*

*Does not change*

*Decreases (somewhat, greatly)*

*I don’t know*

| **Favourable changes after the online resource** | **Runners** | **Non-Runners** | **Knee OA** | **Non**  **knee OA** |
| --- | --- | --- | --- | --- |
| Unfavourable before, who then became favourable after | 90 (5.4) | 40 (4.9) | 87 (7.8) | 43 (3.1) |
| Unfavourable before, who then became neutral after | 78 (4.7) | 44 (5.4) | 65 (5.8) | 57 (4.2) |
| Neutral before, who then became favourable after | 251 (15.1) | 132 (16.1) | 175 (15.6) | 208 (15.2) |
| "I don't know" before, who became favourable after | 55 (3.3) | 30 (3.7) | 63 (5.6) | 22 (1.6) |

| **Unfavourable changes after the online resource** | **Runners** | **Non-Runners** | **Knee OA** | **Non**  **knee OA** |
| --- | --- | --- | --- | --- |
| Favourable before, who then became unfavourable after | 23 (1.4) | 17 (2.1) | 17 (1.5) | 23 (1.7) |
| Favourable before, who then became neutral after | 56 (3.4) | 32 (3.9) | 43 (3.8) | 45 (3.3) |
| Neutral before, who then became unfavourable after | 44 (2.6) | 28 (3.4) | 33 (3) | 39 (2.9) |
| "I don't know" before, who became unfavourable after | 15 (0.9) | 6 (0.7) | 18 (1.6) | 3 (0.2) |

|  | **Runners** | **Non-Runners** | **Knee OA** | **Non**  **knee OA** |
| --- | --- | --- | --- | --- |
| Did not change after the online resource | 1014 (55.7) | 476 (54.5) | 577 (46.9) | 913 (62.4) |
| Changed to “I don’t know” after | 7 (0.4) | 5 (0.6) | 6 (0.5) | 6 (0.4) |

**Q5. Running long distances (such as marathons and ultra-marathons) ____________ the risk of getting knee OA.**

*Increases (greatly, somewhat)*

*Does not change*

*Decreases (somewhat, greatly)*

*I don’t know*

| **Favourable changes after the online resource** | **Runners** | **Non-Runners** | **Knee OA** | **Non**  **knee OA** |
| --- | --- | --- | --- | --- |
| Unfavourable before, who then became favourable after | 29 (1.7) | 11 (1.3) | 24 (2.1) | 16 (1.2) |
| Unfavourable before, who then became neutral after | 72 (4.3) | 26 (3.2) | 55 (4.9) | 43 (3.1) |
| Neutral before, who then became favourable after | 32 (1.9) | 13 (1.6) | 17 (1.5) | 28 (2.1) |
| "I don't know" before, who became favourable after | 8 (0.5) | 8 (1) | 10 (0.9) | 6 (0.4) |

| **Unfavourable changes after the online resource** | **Runners** | **Non-Runners** | **Knee OA** | **Non**  **knee OA** |
| --- | --- | --- | --- | --- |
| Favourable before, who then became unfavourable after | 73 (4.4) | 41 (5) | 42 (3.8) | 72 (5.3) |
| Favourable before, who then became neutral after | 27 (1.6) | 7 (0.9) | 21 (1.9) | 13 (1) |
| Neutral before, who then became unfavourable after | 210 (12.6) | 124 (15.1) | 135 (12.1) | 199 (14.6) |
| "I don't know" before, who became unfavourable after | 109 (6.5) | 47 (5.7) | 92 (8.2) | 64 (4.7) |

|  | **Runners** | **Non-Runners** | **Knee OA** | **Non**  **knee OA** |
| --- | --- | --- | --- | --- |
| Did not change after the online resource | 1063 (58.4) | 523 (59.8) | 685 (55.7) | 901 (61.5) |
| Changed to “I don’t know” after | 17 (1) | 11 (1.3) | 12 (1.1) | 16 (1.2) |

**Q6. It is _________ for a non-runner with knee OA to start a running program if they don’t have symptoms before or after they go running.**

*Appropriate (very, somewhat)*

*Neither appropriate nor inappropriate*

*Inappropriate (somewhat, very)*

*I don’t know*

| **Favourable changes after the online resource** | **Runners** | **Non-Runners** | **Knee OA** | **Non**  **knee OA** |
| --- | --- | --- | --- | --- |
| Unfavourable before, who then became favourable after | 127 (7.6) | 90 (11) | 126 (11.3) | 91 (6.7) |
| Unfavourable before, who then became neutral after | 24 (1.4) | 12 (1.5) | 19 (1.7) | 17 (1.2) |
| Neutral before, who then became favourable after | 145 (8.7) | 82 (10) | 111 (9.9) | 116 (8.5) |
| "I don't know" before, who became favourable after | 111 (6.7) | 48 (5.9) | 127 (11.3) | 32 (2.3) |

| **Unfavourable changes after the online resource** | **Runners** | **Non-Runners** | **Knee OA** | **Non**  **knee OA** |
| --- | --- | --- | --- | --- |
| Favourable before, who then became unfavourable after | 15 (0.9) | 4 (0.5) | 9 (0.8) | 10 (0.7) |
| Favourable before, who then became neutral after | 16 (1) | 12 (1.5) | 13 (1.2) | 15 (1.1) |
| Neutral before, who then became unfavourable after | 5 (0.3) | 1 (0.1) | 6 (0.5) | 0 (0) |
| "I don't know" before, who became unfavourable after | 4 (0.2) | 1 (0.1) | 5 (0.5) | 0 (0) |

|  | **Runners** | **Non-Runners** | **Knee OA** | **Non**  **knee OA** |
| --- | --- | --- | --- | --- |
| Did not change after the online resource | 1210 (66.5) | 561 (64.2) | 689 (56) | 1082 (73.9) |
| Changed to “I don’t know” after | 5 (0.3) | 5 (0.6) | 6 (0.5) | 4 (0.3) |

**Q7. People with knee OA who continue to run will __________ their risk of getting more knee pain.**

*Increases (greatly, somewhat)*

*Does not change*

*Decreases (somewhat, greatly)*

*I don’t know*

| **Favourable changes after the online resource** | **Runners** | **Non-Runners** | **Knee OA** | **Non**  **knee OA** |
| --- | --- | --- | --- | --- |
| Unfavourable before, who then became favourable after | 169 (10.1) | 93 (11.3) | 151 (13.5) | 111 (8.1) |
| Unfavourable before, who then became neutral after | 183 (11) | 104 (12.7) | 164 (14.7) | 123 (9) |
| Neutral before, who then became favourable after | 115 (6.9) | 84 (10.2) | 72 (6.4) | 127 (9.3) |
| "I don't know" before, who became favourable after | 60 (3.6) | 21 (2.6) | 60 (5.4) | 21 (1.5) |

| **Unfavourable changes after the online resource** | **Runners** | **Non-Runners** | **Knee OA** | **Non**  **knee OA** |
| --- | --- | --- | --- | --- |
| Favourable before, who then became unfavourable after | 8 (0.5) | 7 (0.9) | 9 (0.8) | 6 (0.4) |
| Favourable before, who then became neutral after | 77 (4.6) | 32 (3.9) | 39 (3.5) | 70 (5.2) |
| Neutral before, who then became unfavourable after | 5 (0.3) | 3 (0.4) | 6 (0.5) | 2 (0.2) |
| "I don't know" before, who became unfavourable after | 8 (0.5) | 5 (0.6) | 11 (1) | 2 (0.2) |

|  | **Runners** | **Non-Runners** | **Knee OA** | **Non**  **knee OA** |
| --- | --- | --- | --- | --- |
| Did not change after the online resource | 954 (52.4) | 442 (50.6) | 518 (42.1) | 878 (60) |
| Changed to “I don’t know” after | 21 (1.3) | 7 (0.9) | 18 (1.6) | 10 (0.7) |

**Q8. People with knee OA who keep running regularly will ____________ the need for joint replacement surgery.**

*Increases (greatly, somewhat)*

*Does not change*

*Decreases (somewhat, greatly)*

*I don’t know*

| **Favourable changes after the online resource** | **Runners** | **Non-Runners** | **Knee OA** | **Non**  **knee OA** |
| --- | --- | --- | --- | --- |
| Unfavourable before, who then became favourable after | 174 (10.4) | 91 (11.1) | 170 (15.2) | 95 (6.9) |
| Unfavourable before, who then became neutral after | 115 (6.9) | 48 (5.9) | 99 (8.8) | 64 (4.7) |
| Neutral before, who then became favourable after | 195 (11.7) | 106 (12.9) | 120 (10.7) | 181 (13.2) |
| "I don't know" before, who became favourable after | 103 (6.2) | 43 (5.2) | 101 (9) | 45 (3.3) |

| **Unfavourable changes after the online resource** | **Runners** | **Non-Runners** | **Knee OA** | **Non**  **knee OA** |
| --- | --- | --- | --- | --- |
| Favourable before, who then became unfavourable after | 1 (0.1) | 6 (0.7) | 2 (0.2) | 5 (0.4) |
| Favourable before, who then became neutral after | 39 (2.3) | 18 (2.2) | 21 (1.9) | 36 (2.6) |
| Neutral before, who then became unfavourable after | 7 (0.4) | 2 (0.2) | 4 (0.4) | 5 (0.4) |
| "I don't know" before, who became unfavourable after | 9 (0.5) | 8 (1) | 13 (1.2) | 4 (0.3) |

|  | **Runners** | **Non-Runners** | **Knee OA** | **Non**  **knee OA** |
| --- | --- | --- | --- | --- |
| Did not change after the online resource | 960 (52.8) | 469 (53.7) | 521 (42.4) | 908 (62) |
| Changed to “I don’t know” after | 15 (0.9) | 14 (1.7) | 18 (1.6) | 11 (0.8) |

**Q9. It is _________ for runners who have knee OA to continue if they don’t have symptoms before or after they go running.**

*Appropriate (very, somewhat)*

*Neither appropriate nor inappropriate*

*Inappropriate (somewhat, very)*

*I don’t know*

| **Favourable changes after the online resource** | **Runners** | **Non-Runners** | **Knee OA** | **Non**  **knee OA** |
| --- | --- | --- | --- | --- |
| Unfavourable before, who then became favourable after | 72 (4.3) | 43 (5.2) | 80 (7.1) | 35 (2.6) |
| Unfavourable before, who then became neutral after | 10 (0.6) | 2 (0.2) | 8 (0.7) | 4 (0.3) |
| Neutral before, who then became favourable after | 102 (6.1) | 70 (8.5) | 100 (8.9) | 72 (5.3) |
| "I don't know" before, who became favourable after | 79 (4.7) | 29 (3.5) | 89 (8) | 19 (1.4) |

| **Unfavourable changes after the online resource** | **Runners** | **Non-Runners** | **Knee OA** | **Non**  **knee OA** |
| --- | --- | --- | --- | --- |
| Favourable before, who then became unfavourable after | 9 (0.5) | 3 (0.4) | 8 (0.7) | 4 (0.3) |
| Favourable before, who then became neutral after | 7 (0.4) | 12 (1.5) | 11 (1) | 8 (0.6) |
| Neutral before, who then became unfavourable after | 1 (0.1) | 3 (0.4) | 4 (0.4) | 0 (0) |
| "I don't know" before, who became unfavourable after | 3 (0.2) | 1 (0.1) | 3 (0.3) | 1 (0.1) |

|  | **Runners** | **Non-Runners** | **Knee OA** | **Non**  **knee OA** |
| --- | --- | --- | --- | --- |
| Did not change after the online resource | 1374 (75.5) | 652 (74.6) | 806 (65.5) | 1220 (83.3) |
| Changed to “I don’t know” after | 3 (0.2) | 2 (0.2) | 2 (0.2) | 3 (0.2) |
